# Anti-Hair Loss Activity of Healthy Human Scalp-Derived *Staphylococcus capitis* KMH304 Ferment Filtrate in Human Hair-Follicle Dermal Papilla and Keratinocyte Cells

**DOI:** 10.3390/microorganisms14040929

**Published:** 2026-04-20

**Authors:** Hye-Young Yoo, Tae Geun Gil, Na-Rin Kim, Hye-Won Lee, Seoyoung Choi, Sung-Jun Choi, Sung-Ha Park, Byoung-Jun Park

**Affiliations:** 1 Skin & Natural Products Lab, Kolmar Korea Co., Ltd., Seoul 06800, Republic of Korea; hyeyoung_y@kolmar.co.kr (H.-Y.Y.);; 2 Innovative Drug Discovery R&D Institute, BIO-Pharmaceutical Research Center, HK inno.N Co., Ltd., Pangyo 13453, Republic of Korea; 3 Division of R&D Strategy, HK inno.N Co., Ltd., Pangyo 13453, Republic of Korea

**Keywords:** *Staphylococcus capitis*, human scalp-derived microbes, anti-hair loss, anti-aging, longevity, scalp barrier, antioxidant

## Abstract

Scalp microbes are recognized as contributors to hair loss by influencing scalp homeostasis and hair growth. However, the in vitro anti-hair loss activity of microbial culture media derived from healthy scalps remains unclear. In this study, resident microbes from 20 Korean participants with healthy scalps and hair were isolated, and *Staphylococcus capitis* was used to produce *S. capitis* ferment filtrate (SCFF). SCFF anti-hair loss activity was evaluated in human follicle dermal papilla cells (HFDPCs) and human adult low-Calcium High-Temperature (HaCaT) keratinocytes via proliferation assays, qPCR, immunocytochemistry, and SA-β-gal staining at 250–1000 μg/mL. SCFF increased cell density after 48 h in a concentration-dependent manner. In HFDPCs, SCFF controlled growth (KGF, IGF-1, and HGF) and androgen (AR and TGF-β2) factors, regulating key mRNAs for hair growth. SCFF mitigated scalp and hair aging by promoting sirtuins 1 and 7 and collagen type 13, while suppressing p21 and X-Gal staining. In HaCaT cells, SCFF exhibited a scalp barrier-strengthening effect by significantly increasing filaggrin and involucrin levels. It suppressed reactive oxidative stress and exhibited DPPH and ABTS radical scavenging activity. These results suggest that SCFF may modulate key pathways associated with hair loss by promoting scalp and hair anti-aging, barrier strengthening, enhancing antioxidant activity, and supporting hair growth.

## 1. Introduction

Hair loss (alopecia), a phenomenon characterized by hair thinning and reduced density, is classified as androgenetic alopecia (AGA), age-related hair loss, inflammatory alopecia, or alopecia areata, depending on whether it is hormonal or non-hormonal [1]. AGA is driven by heightened sensitivity to testosterone and its active metabolite, dihydrotestosterone. Non-hormonal alopecia is mainly caused by endogenous factors such as genetics and aging; however, external environmental factors such as scalp inflammation, living environment, and psychological factors are also involved [2]. Hair greatly affects an individual’s self-confidence and appearance; therefore, many people invest considerable effort in managing and alleviating hair loss [3]. Previously, general treatments and prevention methods relied on chemical ingredients such as minoxidil (MXD) [4] and finasteride [5], and various cosmetic ingredients such as plant extracts and synthetic ingredients [6,7] have been widely used. However, as the scalp microbiome has recently emerged as a significant factor in scalp and hair health [8,9,10], studies on the correlation between microorganisms and hair loss are increasing [11,12]. Generally, the scalp (skin) has different microbiomes depending on its condition; however, *Staphylococcus* (Firmicutes) is predominantly present in all areas, regardless of whether they are dry or moist [13,14]. The abundance of *Staphylococcus* varies according to the type of hair loss and follicle condition: (1) alopecia areata, 27.4% vs. 32.6% in healthy controls; and (2) AGA, 14% vs. 12% in healthy controls [15]. Nevertheless, most current research has primarily focused on *Staphylococcus epidermis* or *S. aureus* [16,17]; research on the role and effects of *Staphylococcus capitis* on the scalp (skin) or hair loss is currently lacking.

Human follicle dermal papilla cells (HFDPCs) are mesenchymal cells that play a central role in regulating hair growth and cycling and are widely used as an in vitro model to investigate hair-related mechanisms [18,19]. Hair follicle development is regulated by various representative signaling factors such as epidermal growth factor (EGF), insulin-like growth factor-1 (IGF-1), hepatocyte growth factor (HGF), keratinocyte growth factor (KGF), vascular endothelial growth factor (VEGF), androgen receptor (AR), and transforming growth factor-beta (TGF-β). Among these, HGF and KGF (also known as FGF7) not only regulate the interaction between epithelial keratinocytes and HFDPCs but also play a major role in follicular growth and differentiation. Additionally, IGF-1 is essential for hair growth regulation and the transition to the anagen phase [20].

Furthermore, hair loss is related to increased AR, which is more abundant in the dermal papilla cells of AGA scalps and is involved in promoting cellular aging. AR overexpression and signaling are also associated with the expression of TGF-β [21]. TGF-β1 blocks the anagen phase, is the main inducer of the catagen phase in the hair cell cycle and contributes to hair loss prevention when reduced, while TGF-β2 induces premature hair follicle regression [3,22,23]. Furthermore, several studies have shown that scalp aging contributes to hair loss by slowing the proliferation of HFDPCs in balding scalp tissue and altering cell morphology and the expression of age-related factors. Cellular aging in HFDPCs is characterized by increased p16 and p21 expression, altered sirtuin expression (e.g., SIRT3 and SIRT7), and changes in extracellular matrix (ECM) collagen [23,24]. Oxidative stress is a prominent aging-related factor, and individuals with hair loss show a reduced capacity to manage it [25,26]. Oxidative stress causes barrier weakening, leading to symptoms such as flaking and dryness [27]. Therefore, filaggrin, which is essential for barrier formation and maintenance, and tight junctions (such as involucrin and loricrin), which are involved in cell–cell connections, are important for strengthening the scalp and hair [28,29].

A complete understanding of the interactions between and the influence of scalp microbes on hair follicles is lacking. Consequently, the lack of functional validation of healthy scalp microbes has restricted the development of microbe-derived cosmetic ingredients to a few strains, such as *Lactobacillus*. Overall, although comparative studies on the scalp microbiome of individuals with and without hair loss exist, research on the isolation and identification of scalp microbes, their composition, and their potential to alleviate hair loss is lacking. Therefore, this study aimed to characterize the microbial composition of the healthy scalp and to identify the beneficial roles and effects of these microbes. Furthermore, because of the risk of bacterial contamination in cosmetics, the development and application of live microorganisms in cosmetic ingredients are currently prohibited in Korea. Therefore, we optimized the culture conditions for *S. capitis*, a representative strain isolated from healthy individuals, prepared the final culture supernatant through centrifugation and filtration to reduce contamination risk, and utilized it as an active cosmetic ingredient. Because hair loss and scalp health are influenced by a complex interplay of extrinsic and intrinsic factors, this study aimed to investigate the microbial composition of healthy scalps and to evaluate the potential of *Staphylococcus capitis* ferment filtrate (SCFF) as a novel cosmetic ingredient for scalp health by regulating key markers associated with hair growth, scalp and hair anti-aging, antioxidant activity, and scalp barrier function.

## 2. Materials and Methods

### 2.1. Study Design and Sample Collection

To analyze scalp microbiome composition, 20 Korean participants (10 male and 10 female), aged 20–65 years (mean 33.2 years), living in Seoul and surrounding areas, Republic of Korea, were recruited by Kolmar Korea (Seoul, Republic of Korea). This study was approved by the Kolmar Korea Institutional Review Board (N01-2022022-001-HR) and written informed consent was obtained from all participants. The researchers evaluated participants using the Basic and Specific classification standard, and healthy participants (HPs) were classified as basic types (L, M0, C0). The participants were prohibited from using any scalp products, such as shampoos, for 24 h before the collection of microbial samples. The exclusion criteria, scalp sample collection method, and sampling site were the same as those used in previous studies [30,31]. Similarly, sampling was performed for 3 min in the parietal scalp and vertex regions (15–25 cm^2^) using sterile cotton swabs, in a controlled environment maintained at 24 ± 2 °C and 50 ± 5% humidity. The collected scalp-microbial samples were isolated immediately. Microorganisms from each participant were isolated, their abundance was quantified using 16s rRNA analysis, major taxa were identified, and their potential hair loss-alleviating effects were evaluated.

### 2.2. Microbial Ferment Filtrate Preparation

#### 2.2.1. Microbe Isolation, Identification, and Selection

Scalp microbiome samples were processed to isolate the microbial strains under controlled conditions in the following order: (1) Samples were pre-incubated in an anaerobic chamber for 1 h to enhance strain diversity. (2) Serial dilutions (1/4–1/8 of the original concentration) were prepared and plated on MRS and TSB agars. (3) Thereafter, the plates were incubated at 37 °C for 3 days under both aerobic and anaerobic conditions. (4) Morphologically distinct colonies were selected based on their size, shape, and color. The selected colonies were subjected to two rounds of streaking to ensure purity. (5) Next, the purified colonies underwent 16s rRNA sequencing for taxonomic identification (Macrogen Inc., Seoul, Republic of Korea). DNA extraction was performed using an Axen™ Total DNA Mini Kit (Macrogen Inc., Seoul, Republic of Korea), and PCR amplification was conducted using universal primers. The amplified sequences were compared with reference databases (NCBI BLAST) to confirm strain identity. A total of 599 strains were isolated, and the key healthy-participant strain was further analyzed for its potential application in hair loss alleviation. A schematic of this process is provided in Figure S1.

#### 2.2.2. Preparation of SCFF

The selected strain (*Staphylococcus capitis* KMH304; 16286BP, KCTC) was isolated from the scalp of a healthy individual in their 20s and was stored at −80 °C in a glycerol-supplemented cryopreservation medium (20%, *v*/*v* glycerol). *S. capitis* KMH304 was cultured under optimized conditions to ensure its viability and scalability for potential applications. The culture medium (LCL015; Kolmar Korea, Seoul, Republic of Korea) comprised d-glucose, yeast extract, soy peptone, proteose peptone, casein peptone, and salts such as sodium acetate and magnesium sulfate, and was sterilized at 121 °C for 20 min and stored at 4 °C until use. To prepare the stock culture, a 2% (*v*/*v*) inoculum of *S. capitis* KMH304 was cultivated in 10 mL of basal medium at 37 °C with shaking at 200 rpm overnight. For the main culture, 3 mL of the stock culture was inoculated into 300 mL of basal medium in 500 mL baffled flasks and incubated at 37 °C with shaking at 200 rpm for 5–9 h. After fermentation, the cells were separated from the supernatant by centrifugation at 7871× *g* for 10 min at 4 °C. The resulting supernatant was then filtered using a 0.22 μm pore size filter (431118; Corning Life Science, Corning, NY, USA) to completely remove microorganisms. The filtered *S. capitis* KMH304 cell-free culture supernatant, referred to as *S. capitis* ferment filtrate (SCFF), was collected under sterile conditions and stored for further analysis.

### 2.3. In Vitro Analysis

#### 2.3.1. Cell Culture and Treatment

HFDPCs (C-12071; Promocell, Heidelberg, Germany) were cultured in Follicle Dermal Papilla Cell Growth Medium (C-26501; Promocell) supplemented with 1% antibiotic–antimycotic (Gibco; Thermo Fisher Scientific, Waltham, MA, USA). For the in vitro experiments, cells were seeded in Dulbecco’s modified Eagle’s medium (DMEM; SH 30243.01; Hyclone, Logan, UT, USA) supplemented with 5% fetal bovine serum (FBS; 16000-044; Gibco; Thermo Fisher Scientific) and 1% antibiotic–antimycotic (15240-062; Gibco; Thermo Fisher Scientific) [32]. Before treatment, DMEM was replaced with DMEM containing 1% antibiotic–antimycotic for 24 h of serum starvation. The human keratinocyte cell line, Human adult low Calcium High Temperature (HaCaT; Kyung Hee University, Yongin-si, Republic of Korea), was cultured in Iscove’s Modified Dulbecco’s Medium (IMDM; 12440-053; Thermo Fisher Scientific) supplemented with 10% FBS and 1% antibiotic–antimycotic. FBS-free medium was used in all experiments. All experiments were performed in triplicate.

#### 2.3.2. Cell Viability and Proliferation Assay

Cell viability assays were performed using a cell counting kit-8 (CCK-8; CK04-13; Dojindo Laboratories, Kumamoto, Japan) according to the manufacturer’s protocol. HFDPCs and HaCaT cells were seeded at 5 × 10^3^ and 1 × 10^4^ cells/well, respectively, in 96-well plates and incubated for 24 h, after which they were treated with varying concentrations of SCFF (50–5000 μg/mL) for 22 h. Subsequently, 10 µL WST-8 solution was added to each well and incubated for 2 h, and cell viability was measured at an absorbance of 450 nm using a microplate reader (Varioskan LUX, Thermo Fisher Scientific).

The cellular proliferation assay was conducted with HFDPCs and HaCaT cells seeded at 4 × 10^4^ and 6 × 10^4^ cells/well, respectively, in 12-well plates and incubated for 24 h. Thereafter, the cells were treated for 48 h with SCFF at varying concentrations (250–1000 μg/mL), after which images were captured using an Olympus CKX53SF microscope (Olympus Corporation, Tokyo, Japan) with a 10× objective.

#### 2.3.3. SA-β-Gal Assay

HFDPCs were seeded at 4 × 10^4^ cells/well in 12-well plates and incubated for 24 h. After treatment with SCFF for 24 h, SA-β-gal activity was measured using a β-Galactosidase Staining Kit (ab65351, Senescence; Abcam, Cambridge, UK) according to the manufacturer’s protocol. Young cells were used at passage 6, and aged senescent cells were used at passage 16. The proportion of SA-β-gal-positive cells was determined by counting blue-stained cells relative to the total cell number in images acquired using an Olympus CKX53SF microscope (Olympus Corporation) with a 10× objective.

#### 2.3.4. Quantitative Real-Time Polymerase Chain Reaction (qPCR)

HFDPCs and HaCaT cells were seeded at 2 × 10^5^ and 6 × 10^5^ cells/well, respectively, in 6-well plates and incubated for 24 h to reach 80–90% confluency. Subsequently, the cells were treated for 24 h with varying concentrations of SCFF (250–1000 μg/mL). For HFDPCs, the negative (NC) and positive (PC) controls comprised serum-free IMEM and MXD and fisetin (Fis), respectively, while those for HaCaT cells were serum-free DMEM and *N*-acetyl-d-glucosamine, respectively. Total RNA was extracted using Trizol reagent (1559601; Invitrogen, Waltham, MA, USA), and cDNA was synthesized using a cDNA Synthesis Kit (ET21100; PhileKorea, Seoul, Republic of Korea) with 2 μg of RNA. qPCR was performed using SYBR Green PCR Master Mix (QPK-201; Toyobo, Osaka, Japan) on an Applied Biosystems Real-Time PCR System (Thermo Fisher Scientific). mRNA expression levels were calculated using the 2^−ΔΔCt^ method and normalized to the expression levels of the housekeeping gene glyceraldehyde 3-phosphate dehydrogenase (*GAPDH*). The primers used for amplifying specific genes are listed in Table 1.

#### 2.3.5. Antioxidant Capacity

*2,2-Diphenyl-1-picrylhydrazyl (DPPH) radical scavenging.* After mixing a 0.5 mM DPPH solution with the samples at various concentrations in a 1:1 ratio, the mixture was incubated at room temperature (20 ± 5 °C) for 0.5 h. Thereafter, the absorbance was measured at 540 nm using a microplate reader.

*2,2′-azinobis(3-ethylbenzothiazoline-6-sulfonic acid) diammonium salt (ABTS) radical scavenging.* ABTS (15 mM) and potassium persulfate stock (4.9 mM) were mixed in a 1:1 ratio and reacted at room temperature for 12–16 h to produce a working solution. The working solution was diluted with methanol to obtain an absorbance of 0.7 ± 0.02. The samples were then mixed with the working solution at a 1:9 ratio in a 96-well plate and incubated at room temperature for 7 min. The absorbance was measured at 734 nm using a microplate reader. Distilled water and l-ascorbic acid (Vitamin C) were used as NCs and PCs, respectively. The DPPH and ABTS radical scavenging activities were calculated using the following formula:

(1) $$\left[1-\frac{\mathrm{Sample}\;(\mathrm{Radical absorbance}-\mathrm{Ethanol absorbance})}{\mathrm{Blank}\;(\mathrm{Radical absorbance}-\mathrm{Ethanol absorbance})}\right]\times 100$$

#### 2.3.6. Reactive Oxygen Species Production Inhibition (DCF-DA Staining)

HaCaT cells were seeded at a density of 2 × 10^4^ cells/well in 8-well chamber slides and cultured for 24 h. Next, the cells were treated for 24 h with various concentrations of SCFF (250–1000 μg/mL) and then incubated with 20 μM 2′,7′-dichlorofluorescin diacetate solution (DCF-DA, D6883; Sigma-Aldrich, St. Louis, MO, USA) for 20 min at 37 °C in the dark. After incubation, the cells were washed with PBS to remove excess dye and then treated with 800 μM H_2_O_2_ solution (216761; Sigma-Aldrich) for 1 h at 37 °C in a 5% CO_2_ atmosphere. Fluorescence images were acquired using an Olympus IX83 fluorescence microscope (Olympus Corporation) equipped with a LUCPLFLN 20× objective, and reactive oxygen species (ROS) intensity (green fluorescence) was quantified using cellSens Dimension software 3.1 (Evident Corporation, Tokyo, Japan) according to the following formula:

(2) $$[\frac{\mathrm{Fluorescence intensity of treated group}}{\mathrm{Fluorescence intensity of}\;H_{2}O_{2}\;\mathrm{treated group}}]\times 100$$

### 2.4. Statistical Analysis

Data are expressed as the mean ± standard deviation (SD). Data were analyzed using one-way ANOVA, followed by Dunnett’s test. Statistical significance was considered at **** *p* < 0.0001, *** *p* < 0.001, ** *p* < 0.01, and * *p* < 0.05 compared with the control group. Statistical analyses were performed using GraphPad Prism 10.5.0 (GraphPad Software, Inc., La Jolla, CA, USA).

## 3. Results

### 3.1. Isolation and Identification

Twenty Korean participants were recruited for this study. Their ages were distributed as follows: 8 participants in their 20s, 9 in their 30s, 2 in their 40s, none in their 50s, and 1 in their 60s. The average age of women was 31 years, and that of men was 35.3 years.

**Figure 1:**
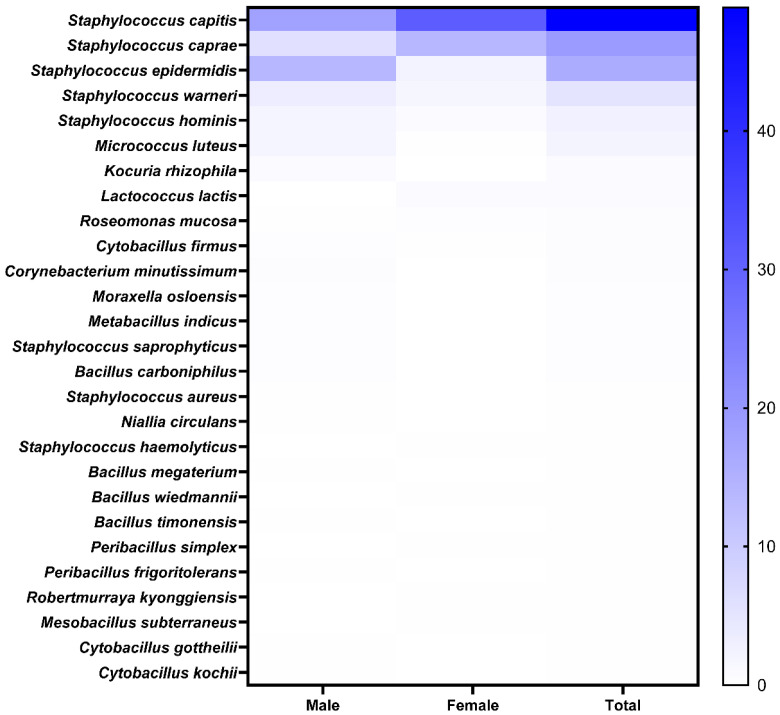
Isolation and identification of scalp strains. Heat map showing prevalence of specific strains in healthy participants.

A total of 599 strains were isolated from scalp samples collected from the participants. After removing duplicate strains and considering their unique accession numbers, 27 strains were identified (Figure 1). The top three strains identified were *S. capitis*, *Staphylococcus caprae*, and *S. epidermidis,* accounting for 48.91, 19.37, and 15.86% of the total strains, respectively. Specifically, *S. capitis* accounted for 17.86% in males and 31.05% in females. Based on these results, *S. capitis*, which was most abundant in HPs (males and females), was selected as the priority strain for further studies. The detailed composition (%), identified strains, and their accession numbers are listed in Table S1.

### 3.2. Effect of SCFF on Cell Viability and Proliferation in HFDPCs and HaCaT Cells

**Figure 2:**
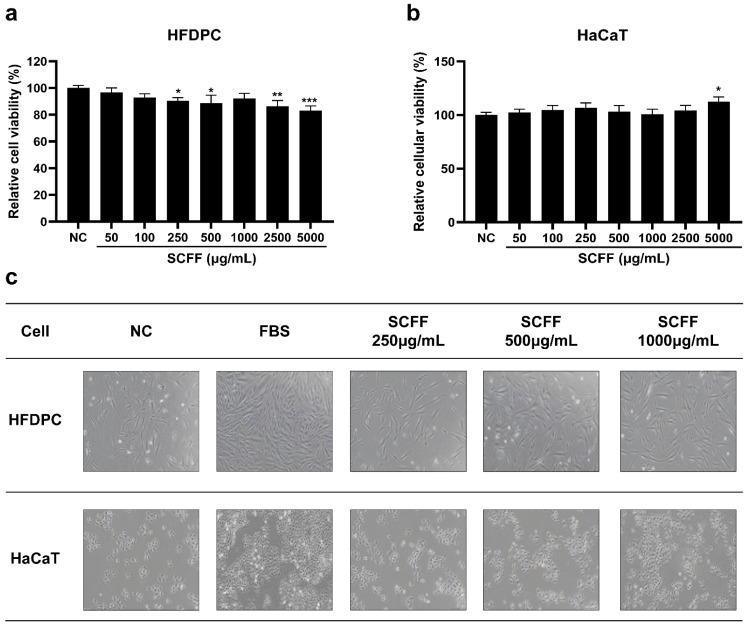
Effect of SCFF on HFDPC and HaCaT cell viability and proliferation. HFDPCs and HaCaT cells were treated with different concentrations of SCFF as indicated for 24 h. (**a**) HFDPC and (**b**) HaCaT viability and proliferation were assessed at various SCFF concentrations (50, 100, 250, 500, 1000, 2500, 5000 μg/mL) using a CCK-8 assay. Values are mean ± SD of three independent experiments. (**a**–**c**) *** *p* < 0.001, ** *p* < 0.01 and * *p* < 0.05 versus NC group (100%). (**c**) Representative images of cellular proliferation activity in HFDPCs and HaCaT cells after 48 h of incubation (*n* = 3; Scale bar = 200 μm). SCFF, *Staphylococcus capitis* KMH304 ferment filtrate; HFDPCs, human follicle dermal papilla cells; NC, negative control.

The optimal experimental concentration and cell proliferation effects of SCFF on HFDPCs and HaCaT cells were determined. A relative viability rate of 90% was used as the criterion for cytotoxicity. In HFDPCs (Figure 2a), SCFF did not exhibit cytotoxicity up to 1000 μg/mL, and in HaCaT cells (Figure 2b), SCFF showed no cytotoxic effects at all tested concentrations for 24 h. Therefore, subsequent experiments in HaCaT cells were conducted within the same range, using the non-toxic concentrations identified in HFDPCs as the reference.

To determine whether SCFF directly affected cell growth, HFDPCs and HaCaT cells were treated with SCFF at 250–1000 μg/mL for 48 h. The density of both cell types improved in a concentration-dependent manner following SCFF treatment compared with that in the NC group. Representative proliferation images of HFDPCs and HaCaT cells are shown in Figure 2c.

### 3.3. Effect of SCFF on Hair Growth-Related mRNA Expression in HFDPCs

**Figure 3:**
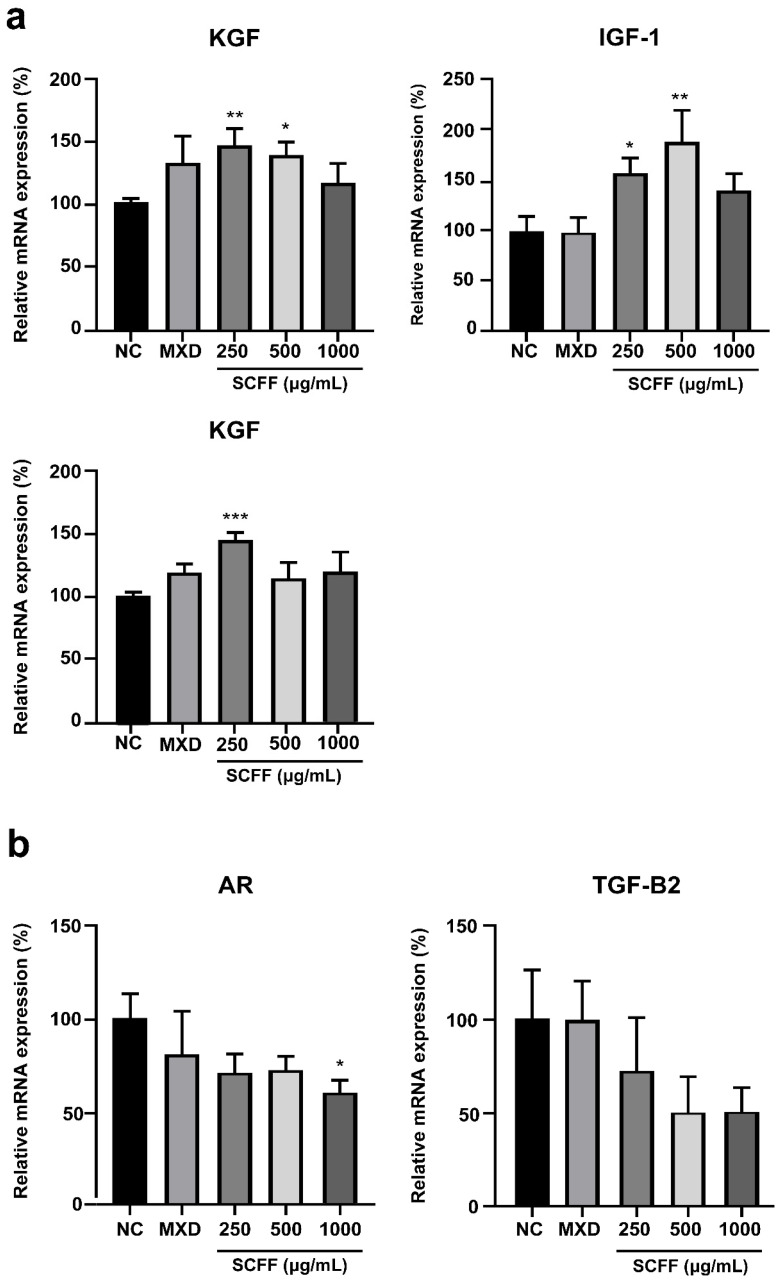
Hair growth-related and AGA-inducing signature gene expression following SCFF treatment in HFDPCs. HFDPCs were treated with different SCFF concentrations for 24 h, and gene expression was evaluated using qPCR. (**a**) Hair growth-related factors (KGF, IGF-1, and HGF). (**b**) AGA-inducing factors (AR and TGF-β2). MXD at 5 μg/mL was used as PC for TGF-β2, and 2.5 μg/mL MXD was used as PC for other markers. Data are mean ± SD of three independent experiments. *** *p* < 0.001, ** *p* < 0.01 and * *p* < 0.05 versus NC group (100%). SCFF, *Staphylococcus capitis* KMH304 ferment filtrate; HFDPCs, human follicle dermal papilla cells; MXD, minoxidil; PC, positive control.

To determine whether SCFF regulates the expression of hair growth-related and AGA inhibition markers in HFDPCs, qPCR was performed (Figure 3). MXD was used as a positive control, as it is reported to stimulate hair growth through its effects on dermal papilla. Compared with the NC group, treatment with SCFF (250–1000 μg/mL) induced the expression of hair growth-related markers (KGF, IGF-1, and HGF) in HFDPCs (Figure 3a). SCFF treatment at concentrations of 250 and 500 μg/mL increased KGF expression by 48% (*p* < 0.01) and 42% (*p* < 0.05), respectively. SCFF treatment increased IGF-1 expression by 58% (*p* < 0.05) at 250 μg/mL, 89% (*p* < 0.01) at 500 μg/mL, and 40% at 1000 μg/mL, respectively. SCFF at 250 μg/mL produced a highly significant 47% increase in HGF expression (*p* < 0.001). MXD (2.5 μg/mL) induced increases of 33% and 20% in KGF and HGF, respectively, while no significant change was observed in IGF-1.

Additionally, SCFF induced a concentration-dependent suppression of AR and TGF-β2 at 250–1000 μg/mL (Figure 3b). Specifically, at a high concentration (1000 μg/mL), AR and TGF-β2 expression decreased by 39% (*p* < 0.05) and 49%, respectively, compared with that in NC. These reductions exceeded the inhibition observed with MXD, which reduced AR and TGF-β2 by 20 and 7%, respectively. These results suggest that SCFF (250–1000 μg/mL) induced hair cell growth by upregulating growth markers and downregulating AGA-inducing markers in HFDPCs.

### 3.4. Effect of SCFF on Scalp- and Hair-Related Anti-Aging mRNA Expression in HFDPCs

**Figure 4:**
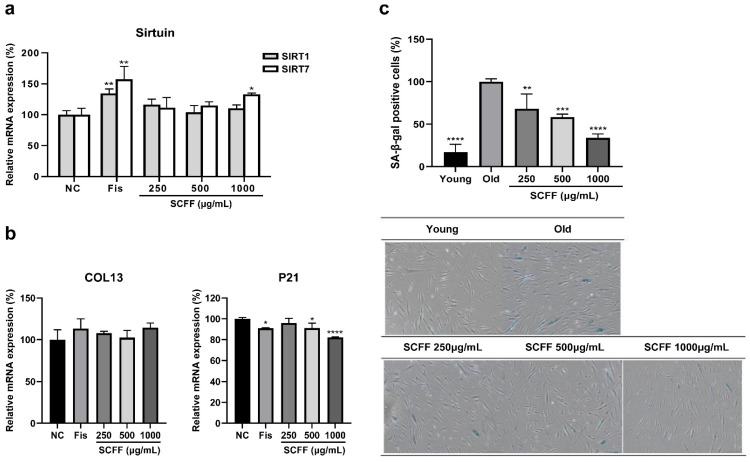
The regulation of cellular senescence by SCFF in HFDPCs. HFDPCs were treated with different concentrations of SCFF for 24 h, followed by analysis using qPCR and SA-β-gal staining. (**a**,**b**) Anti-aging factors (SIRT1, SIRT7, COL13A1, and p21). (**c**) SA-β-gal staining in young (P6) and senescent (P16) passage cells. Quantitative and representative images of SA-β-gal-positive cells compared with senescent cells (Scale bar = 200 μm). For SIRT, 1 μg/mL fisetin (Fis) was used as a positive control (PC), and for COL13 and p21, 1 μg/mL Fis was used as PC. Data are the mean ± SD of three independent experiments. **** *p* < 0.0001, *** *p* < 0.001, ** *p* < 0.01, and * *p <* 0.05 versus the negative control (NC) and the senescent group (100%). SCFF, *Staphylococcus capitis* KMH304 ferment filtrate; HFDPCs, human follicle dermal papilla cells.

Because scalp and hair aging are associated with hair loss, we examined whether SCFF regulates cellular senescence in HFDPCs. The expression levels of sirtuin, collagen, and SA-β-gal were assessed using qPCR (Figure 4). A natural polyphenol with senolytic properties, Fis, was used as the positive control. When treated with SCFF at 250–1000 μg/mL, the expression levels of SIRT1 and SIRT7, representative cellular-longevity genes, increased, with SIRT1 showing up to 16% elevation. However, SIRT 7 showed a more significant increase in a dose-dependent manner, with a maximum enhancement of 33% (*p* < 0.05) (Figure 4a). In addition, the expression level of COL13A1, which is involved in the suppression of cell senescence in HFDPCs, increased by up to 14% compared with that in the NC group and was similar to that of Fis, a representative anti-aging flavonoid (Figure 4b).

The cellular phenotype of aged HFDPCs was assessed by comparing morphological changes between early young passages (P6) and senescent passages (P16), together with evaluating the expression of canonical senescence-associated markers, including β-galactosidase (β-gal) and p21 (Figure 4b,c). Aged HFDPCs exhibited morphological changes, including an enlarged size, flattened morphology, and increased β-gal activity. We observed reduced X-Gal activity in aged SCFF-treated cells. In senescent cells, we observed a significant increase in the number of SA-β-Gal-positive (blue) cells compared with those in young cells. Following SCFF treatment, the number of positive cells was significantly reduced, with a maximum reduction of 66%. Furthermore, after 24 h of treatment with SCFF, p21 mRNA expression was reduced by 9% (*p* < 0.05) at a concentration of 500 μg/mL and by 18% (*p* < 0.0001) at a concentration of 1000 μg/mL, which was higher than the 9% inhibition rate of Fis. These results suggest that SCFF plays a key role in maintaining scalp ECM reinforcement and preventing senescence.

### 3.5. Scalp Barrier and Antioxidant Effect of SCFF in Keratinocytes

**Figure 5:**
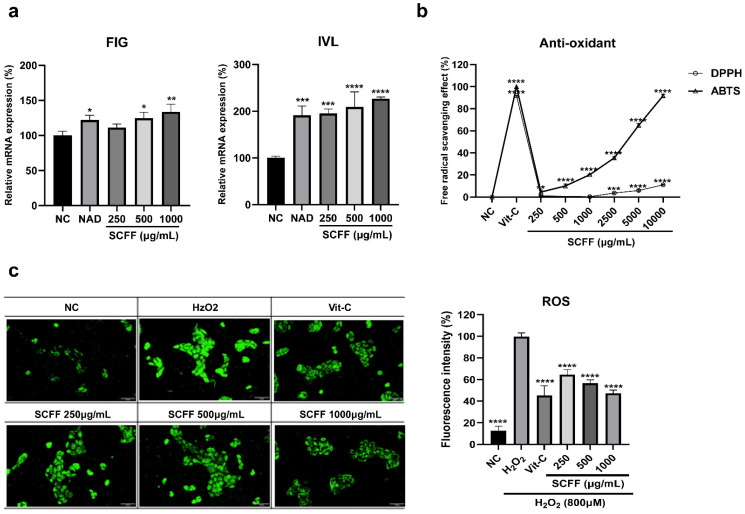
Scalp barrier enhancement and antioxidant activity in keratinocytes (HaCaT cells). (**a**) mRNA expression of barrier components (*FIG* and *IVL*) in HaCaT cells. (**b**) DPPH and ABTS radical-scavenging activity, and (**c**) Reactive oxygen species (ROS) scavenging activity enhanced by H_2_O_2_ at the cellular level (Scale bar = 100 μm). *N*-acetyl-d-glucosamine (5%) was used as the positive control (PC) for barrier function, and 100 µg/mL vitamin C (Vit-C) was used as the PC for the antioxidant assay. Data are the mean ± SD of three independent experiments. **** *p* < 0.0001, *** *p* < 0.001, ** *p* < 0.01, and * *p* < 0.05 versus the NC group (100%) for *FIG*, *IVL*, DPPH, and ABTS. **** *p* < 0.0001 versus the H_2_O_2_ control (100%) for ROS.

Because oxidative stress and barrier damage to the scalp stratum corneum contribute to increased hair loss or shedding, enhancing these two functions in keratinocytes represents an additional key strategy for alleviating hair loss. Therefore, we used HaCaT cells to determine the expression levels of scalp-barrier factors (filaggrin and tight junctions) and to evaluate the degree of ROS production inhibition. The mRNA expression levels of filaggrin (*FIG*) and involucrin (*IVL*), which play roles in barrier reinforcement and functional maintenance, were evaluated following SCFF treatment (Figure 5a). *FIG* expression increased by 11, 25, and 34% following treatment with 250–1000 μg/mL SCFF, while that of IVL rose in a concentration-dependent manner by 95, 109, and 127%, respectively. These effects were greater than those observed with N-acetyl-D-glucosamine (NAD), a precursor of hyaluronic acid and a key component affecting skin hydration and barrier function, which was used as a positive control.

Next, to address hair loss and aging driven by oxidative stress, we evaluated the antioxidant efficacy of SCFF (Figure 5b,c). The DPPH and ABTS free-radical scavenging activities of SCFF were examined across concentrations ranging from 250 to 10,000 μg/mL. DPPH exhibited relatively weak activity, with only 11% scavenging at 10,000 μg/mL. Conversely, ABTS showed a concentration-dependent increase in antioxidant activity, with 10% scavenging at 1000 μg/mL and 92% at 10,000 μg/mL. Furthermore, ROS inhibitory activity was assessed at the cellular level. Treatment with H_2_O_2_ increased ROS levels, whereas SCFF at 250–1000 μg/mL reduced ROS intensity by 35, 43, and 53%, respectively (*p* < 0.0001). DCF fluorescence is an indicator of ROS production. As shown in Figure 5c, after 1 h of exposure to 800 μM H_2_O_2_ in HaCaT cells, DCF fluorescence intensity increased by approximately 87% compared with that in NC. The increase in DCF fluorescence intensity was abolished by SCFF treatment. This inhibition occurred in a concentration-dependent manner, with 250–1000 μg/mL SCFF reducing fluorescence by 35–43%. Vitamin C showed 54% scavenging activity, whereas SCFF showed superior antioxidant efficacy. These results suggest that SCFF may prevent oxidative stress-induced hair loss and aging.

## 4. Discussion

Alopecia significantly affects not only quality of life but also self-confidence, appearance, and depressive symptoms [33]. Accordingly, interest in preventing hair loss is high, and numerous research and development efforts are underway to identify new raw materials and formulate products for this purpose [34,35]. HFDPCs are key regulators of hair follicle formation, regeneration, and growth. However, in vitro studies investigating their role in mitigating hair loss have mainly focused on their responsiveness to various stimuli and related mechanisms. Most previous studies have focused on herbal extracts, peptides, and chemical compounds [18,36].

Recent studies have increasingly focused on the role of the scalp microbiome in maintaining scalp homeostasis and influencing hair-related biological processes [2,37]. However, no study has evaluated the hair loss-preventive effects of the most common strains isolated from individuals with healthy scalps.

In another study conducted in a Korean population, *Staphylococcus* accounted for 47.9% of the scalp microbiome [2]. Together, *Cutibacterium* spp. and *Staphylococcus* spp. make up approximately 90% of the microbiome in healthy scalps [38]. Therefore, in the present study, we determined the strain composition of scalp microbes obtained from 20 Korean individuals. The results revealed that *S. capitis* was the most prevalent species in all participants. We focused on S. capitis, which was found to constitute approximately 50% of the scalp microbiota, indicating its association with scalp health. In an additional analysis comparing the microbial communities of individuals with hair loss and healthy controls, S. capitis was more abundant in healthy subjects (22.96%) than in those with hair loss (17.87%). *S. capitis* KMH304, a strain isolated from a healthy subject in their 20s, was cultured to produce SCFF. *S. capitis* is a commensal species that exists on human skin and resides primarily on the head, arms, and chin [39]. The culture supernatant is straightforward to handle because the removal of microbial cells substantially reduces the risk of contamination. It also has excellent physicochemical stability because the absence of cells greatly reduces the possibility of precipitation [40,41]. In addition, the culture supernatant contains concentrated metabolites, including proteins and peptides produced by the microorganism, can be used immediately without additional extraction, and poses a low risk of skin irritation owing to cell wall component removal [42,43]. Therefore, culture supernatants are expected to be widely used as active cosmetic ingredients owing to their safety, stability, and ease of use. In particular, 87.5% of *S. capitis* strains possessed the ability to form biofilms [44], with biofilm formation being involved in host immunity. Recent research has shown that novel cosmetic active ingredients, such as *Bifidobacterium longum* lysate, provide reactive skin and scalp relief through postbiotics such as *Saccharomyces* and *Lactobacillus* ferment complexes [45,46].

Based on these results, we evaluated the potential of *S. capitis*, a major strain isolated from participants with healthy scalps and hair characteristics, as a new cosmetic ingredient for anti-hair loss and scalp health applications through in vitro culture-based analysis. First, we investigated the cellular proliferation and growth effects of SCFF on HFDPCs and keratinocytes. Because cell proliferation can be used as a surrogate indicator of growth factor upregulation in vitro, we investigated the molecular mechanisms that might promote proliferation in HFDPCs and HaCaT cells. SCFF exhibited proliferative effects in both cell types and significantly upregulated the expression of proliferation-related growth factors IGF-1, HGF, and KGF. IGF-1 and HGF act on progenitor cells through autocrine signaling, strongly promoting cell migration, proliferation, and survival. KGF affects hair growth through cell proliferation, extending the anagen phase, and promoting hair keratin synthesis by inducing the proliferation, migration, and differentiation of keratinocytes. Additionally, TGF-β plays a role in stimulating signaling mechanisms involved in the hair cycle, and several other growth factors act not only on the epidermis but also on other parts of the skin to promote hair growth [47,48]. Because alopecia is characterized by a short anagen phase, promoting hair follicle cell proliferation and growth factor expression with SCFF may help modulate hair loss. Moreover, studies have shown that androgen–AR signaling promotes the premature aging of HFDPCs, which are highly expressed in patients with AGA and contribute to hair follicle miniaturization [49].

Because the pathogenesis of hair loss, including AGA, involves accelerated cellular aging, mitigating cellular aging may represent an additional preventive measure for reducing hair loss and promoting scalp and hair anti-aging. SCFF suppressed cellular aging in a dose-dependent manner through cell cycle factor p16 and SA-β-gal and promoted the expression of scalp collagen (COL13A1) and sirtuins, known as longevity genes, in HFDPCs. When SIRT1 is downregulated, it induces the overexpression of p53, which promotes cellular aging, leading to hair aging [50]. Another sirtuin subtype, SIRT7, is less frequently expressed with aging and is regulated by NFATC1, which is expressed during the catagen phase, a period of hair growth inhibition. SIRT3 and SIRT7 are expressed at higher levels in healthy individuals than in those with AGA [24]. Additionally, collagens 13 and 15 induce spheroid formation, and when blocked, cell senescence is induced [23]. These results indicate that SCFF promotes scalp/hair longevity, enhances scalp collagen integrity, and exerts anti-aging effects on the scalp and hair.

When skin barrier function is impaired, microbial imbalance can occur, contributing to the progression of conditions such as wounds and atopic dermatitis [16,51]. The development of other cells, such as HaCaT cells, in the hair follicle is also essential for hair growth and follicle development, and keratinocytes protect the scalp and hair from external factors in the outermost layer [52,53]. Therefore, enhancing barrier function to protect the hair and scalp from environmental factors, such as pathogenic bacteria and oxidative stress (ROS), represents another strategy for preventing hair loss. In the cosmetic field, numerous studies have demonstrated the efficacy of probiotics, such as *Lactiplantibacillus plantarum* LB244R, and postbiotics, such as *Lacticaseibacillus paracasei* GMNL-653—Heat-Killed, at in vivo, ex vivo, and clinical levels for skin and hair benefits [54,55]. Among the *Staphylococcus* spp., the hair loss-ameliorating effects of *S. epidermidis Cicaria* have been reported in vitro and ex vivo; however, in Human Papilla Cells, strong efficacy on VEGF and FGF-7 was observed only at a 10% concentration, while effects were weak at 0.1% [2]. These findings suggest that research on *Staphylococcus* spp. remains limited, and studies specifically addressing *S. capitis* are currently lacking. Bifida Ferment Lysate (BFL), a fermentation extract of Bifidobacterium—one of the most commonly used probiotics like Staphylococcus—at concentrations ranging from 1 to 5%, demonstrated barrier maintenance efficacy in HaCaT cells, with increases of up to approximately 60% in FIG and 100% in IVL expression. In terms of antioxidant activity, at a concentration of 30%, BFL showed DPPH and ABTS radical scavenging activities of 34.42% and 54.34%, respectively [56]. SCFF showed strong efficacy at relatively low concentrations by promoting barrier-related proteins, including FIG and IVL, scavenging free radicals, and reducing ROS production in HaCaT cells. The ABTS radical scavenging assay operates on the same principle as the DPPH radical scavenging assay; however, ABTS measures the scavenging of cationic radicals, whereas DPPH measures the scavenging of anionic radicals. The difference in the types of radicals and their binding affinities with substrates can lead to variations in the measured radical scavenging activities of extracts, which has been reported previously [57]. Therefore, this may explain the observed differences between the two assays. This suggests that the antioxidant and barrier strengthening properties of SCFF can improve scalp conditions and contribute to hair strength. These findings suggest that SCFF may exert coordinated effects across multiple biological pathways. Specifically, the upregulation of growth factors (KGF, IGF-1, HGF), together with the suppression of androgen-related markers (AR, TGF-β2), and the modulation of aging-associated regulators (SIRT1, SIRT7, and p21), indicating a multifactorial mechanism that may contribute to maintaining hair follicle function at the cellular level.

However, this study has several limitations. First, the number of participants was small and limited to Koreans, which means that the results may reflect microbiome characteristics specific to this population. Second, the mechanisms underlying each SCFF effect were primarily elucidated through mRNA analysis, and more detailed investigations at the protein level are required. Further analysis of the SCFF components and metabolites is necessary to verify the underlying mechanisms. Finally, the identified effective concentrations were applied to cosmetic products such as shampoo to verify their potential for alleviating hair loss ex vivo or clinically. Accordingly, in ongoing follow-up studies, we are exploring the active compounds of SCFF through component analysis to investigate the mechanisms at the protein level. Furthermore, additional scaled-up culture studies are currently underway to further investigate the profile of *S. capitis* and hair loss improvement in human.

Despite these limitations, the data presented here represent a meaningful approach, given the complex interplay of factors affecting scalp and hair biology and the potential for new applications of *S. capitis* culture fluid. SCFF (*S. capitis* KNH304 cultured media), derived from the scalps of healthy individuals, was evaluated at the cellular level. The results suggest that SCFF is a novel cosmetic ingredient that may modulate biological pathways associated with hair growth by promoting cell proliferation, exerting anti-aging effects, and reducing ROS through barrier strengthening.

## 5. Conclusions

**Figure 6:**
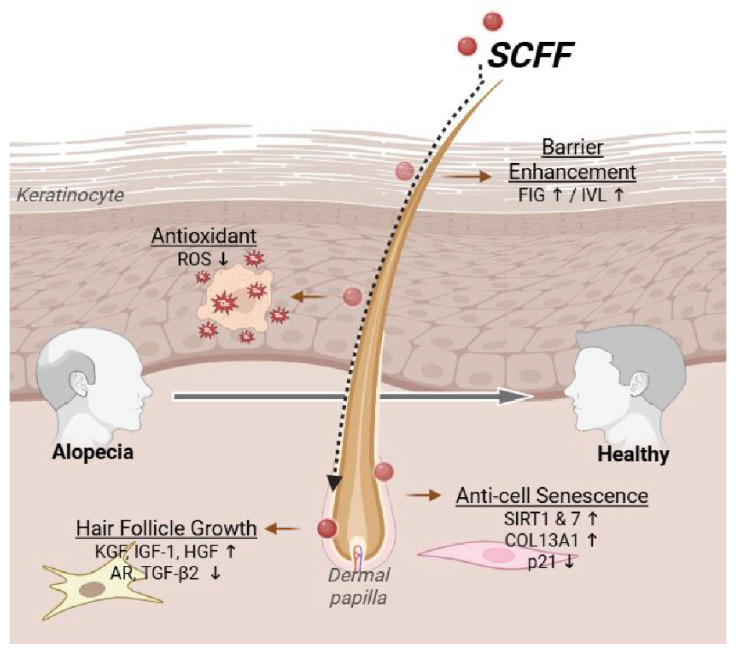
Overall effect of SCFF in HFDPCs and HaCaT cells. In keratinocytes, SCFF enhanced barrier function and antioxidant activity. SCFF not only regulated cellular aging through longevity-, collagen-, and p21-related genes, but also promoted hair growth by increasing the expression of hair-growth–associated factors and suppressing hair loss-inducing factors in HFDPCs. SCFF, *Staphylococcus capitis* KMH304 ferment filtrate; HFDPCs, human follicle dermal papilla cells.

The microbial composition of the scalp is associated with alopecia. Our findings revealed the presence of 27 representative microbial communities. *S. capitis* isolated from a healthy scalp group (aged 20 years) promoted HFDPC proliferation, increased the expression of multiple hair-growth-promoting factors, and inhibited AR activity (Figure 6). Furthermore, SCFF exhibited collagen-enhancing, cell senescence-inhibiting, and longevity-promoting properties. It also improved barrier function and demonstrated antioxidant effects. These results suggest that SCFF has potential as a novel cosmetic ingredient for contributing to anti-hair loss by modulating key pathways associated with hair growth and scalp health.

## Figures and Tables

**Table 1:** Primer sequences used for amplifying specific genes.

Species	Purpose	Primer	Forward	Reverse
Human	Hair follicle growth	KGF	ATCAGGACAGTG GCAGTTGGA	AACATTTCCCCT CCGTTGTGT
		IGF-1	AGGAAGTACATTTGA AGAACG	CCTGCGGTGGCATGTCA
		HGF	AGAAATGCAGCCAGC ATCAT	CACATGGTCCTGATCCAATC
	AGA inhibition	AR	GGAATTCCTGTGCATGAAA	CGAAGTTCATCAAAGAATT
		TGF-β2	ATCTAGGGTGGAAATGGATACACG	TGGTTAGAGGTTCTAAATCT
	Scalp/Hair anti-aging	SIRT1	TGTTTCCTGTGGGATACCTGA	TGAAGAATGGTCTTGGGTCTTT
		SIRT7	TGGAGTGTGGACACTGCTTCAG	CCGTCACAGTTCTGAGACACCA
		COL13A1	TGGAGAACAGGGACCAGATGGC	GATCTCCTGGAGAGCCTCATTG
		p21	AGGTGGACCTGGAGACTCTCAG	TCCTCTTGGAGAAGATCAGCCG
	Scalp barrier	Filaggrin	TGAGGCATACCCAGAGGACT	CTGTATCGCGGTGAGAGGAT
		Involucrin	TCCACTTATTTCGGGTCCGC	GGGGTTGGCACTGGACAATA
	GAPDH		GTCTCCTCTGACTTCAACAGCG	ACCACCCTGTTGCTGTAGCCAA

## Data Availability

The original contributions presented in this study are included in the article/Supplementary Material. Further inquiries can be directed to the corresponding author.

## Supplementary Materials

The following supporting information can be downloaded from https://www.mdpi.com/article/10.3390/microorganisms14040929/s1, Figure S1: Scalp strain isolation and identification, Table S1: Identified strains with detailed composition ratios (%) and accession numbers.

## Abbreviations

The following abbreviations are used in this manuscript:

KGF	Keratinocyte growth factor
IGF-1	Insulin-like growth factor-1
HGF	Hepatocyte growth factor
AR	Androgen receptor
TGF-β	Transforming growth factor-β
FGF7	Fibroblast growth factor 7
NC	Negative control
PC	Positive control
HP	Healthy participants
ECM	Extracellular Matrix
Fis	Fisetin
MXD	Minoxidil
AGA	Androgenetic alopecia
DMEM	Dulbecco’s modified Eagle’s medium
EGF	Epidermal growth factor
HFDPCs	Human follicle dermal papilla cells
HaCaT	Human adult low Calcium High Temperature
ROS	Reactive oxygen species
SD	Standard deviation
VEGF	Vascular endothelial growth factor
