# Supplementary material for: Anti-Hair Loss Activity of Healthy Human Scalp-Derived Staphylococcus capitis KMH304 Ferment Filtrate in Human Hair-Follicle Dermal Papilla and Keratinocyte Cells
Source: Microorganisms. 2026 Apr 20;14(4):929. doi: 10.3390/microorganisms14040929 (PMC13119399; doi:10.3390/microorganisms14040929)
Supplement: Supplementary file 1 [file microorganisms-14-00929-s001.zip › microorganisms-4199421-supplementary.pdf]

Supplementary Materials

Supplementary Figures

Pre-Incubation and Spreading
(Anaerobic chamber, 1 h, MRS/TSB agar)

↓

First Activation [Streaking]
(Securing morphologically stable single colonies)

↓

Second Activation [Streaking]
(Securing final single colonies for ID analysis and pre-RCB preparation)

↓

16s rRNA Sequencing Analysis [Identification]

↓

Inoculation of Colonies for pre-RCB Preparation
(Media cultivation for pre-RCB)

↓

pre-RCB Preparation and Storage
(Frozen storage at -80 °C)

Figure S1. Scalp-strain isolation and identification. The process includes pre-incubation in an anaerobic chamber for 1 h, optimal dilution (1/4–1/8), smear experiments, two rounds of streaking for colony purification, 16s rRNA sequencing for strain identification, and pre-RCB preparation, followed by frozen storage at -80 °C. Each step is designed to enhance strain diversity and ensure purity.

Supplementary tables

Table S1. Identified strains with detailed composition ratios (%) and accession numbers.

No.	Strain	Accession number	Male	Female	Total
1	*Staphylococcus capitis*	NR_113348.1, NR_027519.1	17.86	31.05	48.91
2	*Staphylococcus caprae*	NR_024665.1	5.68	13.69	19.37
3	*Staphylococcus epidermidis*	NR_113957.1, NR_036904.1, LN681574.1	13.69	2.17	15.86
4	*Staphylococcus warneri*	NR_025922.1	3.34	1.67	5.01
5	*Staphylococcus hominis*	NR_036956.1	1.84	0.67	2.50
6	*Micrococcus luteus*	LN681571.1, NR_075062.2	1.84	0.17	2.00
7	*Kocuria rhizophila*	NR_026452.1	0.83	0.00	0.83
8	*Lactococcus lactis*	NR_113958.1, NR_040956.1	0.00	0.67	0.67
9	*Roseomonas mucosa*	NR_028857.1	0.17	0.33	0.50
10	*Cytobacillus firmus*	NR_112635.1, NR_025842.1	0.33	0.17	0.50
11	*Corynebacterium minutissimum*	LN831313.1	0.50	0.00	0.50
12	*Moraxella osloensis*	CP014234.1	0.33	0.00	0.33
13	*Metabacillus indicus*	NR_029022.1	0.33	0.00	0.33
14	*Staphylococcus saprophyticus*	AP008934.1	0.33	0.00	0.33
15	*Bacillus carboniphilus*	NR_024690.1	0.33	0.00	0.33
16	*Staphylococcus aureus*	CP011526.1	0.17	0.00	0.17
17	*Niallia circulans*	NR_112632.1, NR_104566.1	0.17	0.00	0.17
18	*Staphylococcus haemolyticus*	NR_113345.1	0.00	0.17	0.17
19	*Bacillus megaterium*	CP009920.1	0.17	0.00	0.17
20	*Bacillus wiedmannii*	KU198626.1	0.00	0.17	0.17
21	*Bacillus timonensis*	NR_133024.1	0.17	0.00	0.17
22	*Peribacillus simplex*	NR_114919.1	0.00	0.17	0.17
23	*Peribacillus frigoritolerans*	NR_117474.1	0.17	0.00	0.17
24	*Robertmurraya kyonggiensis*	NR_132682.1	0.00	0.17	0.17
25	*Mesobacillus subterraneus*	NR_104749.1	0.00	0.17	0.17
26	*Cytobacillus gottheilii*	NR_108491.1	0.17	0.00	0.17
27	*Cytobacillus kochii*	NR_117050.1	0.17	0.00	0.17
